# Does use of the CONSORT Statement impact the completeness of reporting of randomised controlled trials published in medical journals? A Cochrane review^a^

**DOI:** 10.1186/2046-4053-1-60

**Published:** 2012-11-29

**Authors:** Lucy Turner, Larissa Shamseer, Douglas G Altman, Kenneth F Schulz, David Moher

**Affiliations:** 1 Clinical Epidemiology Program, Ottawa Hospital Research Institute, Ottawa, Canada; 2 Centre for Statistics in Medicine, University of Oxford, Oxford, UK; 3 FHI 360 and UNC School of Medicine, Quantitative Sciences, Research Triangle Park, NC, USA; 4 Department of Epidemiology and Community Medicine, University of Ottawa, Ottawa, Canada

**Keywords:** CONSORT, Endorsement, Reporting guideline, Completeness of reporting

## Abstract

**Background:**

The Consolidated Standards of Reporting Trials (CONSORT) Statement is intended to facilitate better reporting of randomised clinical trials (RCTs). A systematic review recently published in the Cochrane Library assesses whether journal endorsement of CONSORT impacts the completeness of reporting of RCTs; those findings are summarised here.

**Methods:**

Evaluations assessing the completeness of reporting of RCTs based on any of 27 outcomes formulated based on the 1996 or 2001 CONSORT checklists were included; two primary comparisons were evaluated. The 27 outcomes were: the 22 items of the 2001 CONSORT checklist, four sub-items describing blinding and a ‘total summary score’ of aggregate items, as reported. Relative risks (RR) and 99% confidence intervals were calculated to determine effect estimates for each outcome across evaluations.

**Results:**

Fifty-three reports describing 50 evaluations of 16,604 RCTs were assessed for adherence to at least one of 27 outcomes. Sixty-nine of 81 meta-analyses show relative benefit from CONSORT endorsement on completeness of reporting. Between endorsing and non-endorsing journals, 25 outcomes are improved with CONSORT endorsement, five of these significantly (α = 0.01). The number of evaluations per meta-analysis was often low with substantial heterogeneity; validity was assessed as low or unclear for many evaluations.

**Conclusions:**

The results of this review suggest that journal endorsement of CONSORT may benefit the completeness of reporting of RCTs they publish. No evidence suggests that endorsement hinders the completeness of RCT reporting. However, despite relative improvements when CONSORT is endorsed by journals, the completeness of reporting of trials remains sub-optimal. Journals are not sending a clear message about endorsement to authors submitting manuscripts for publication. As such, fidelity of endorsement as an ‘intervention’ has been weak to date. Journals need to take further action regarding their endorsement and implementation of CONSORT to facilitate accurate, transparent and complete reporting of trials.

## Background

In the mid-1990s, in response to concerns about the quality of reporting of randomised controlled trials (RCTs), an international group of trialists, statisticians, epidemiologists and biomedical editors developed The CONsolidated Standards Of Reporting Trials (CONSORT) Statement [1], which has been revised and updated twice [2,3], each with a companion explanatory document [4,5]. The Statement is an evidence-based minimum set of recommendations, consisting of a checklist, flow diagram and descriptive text, intended to facilitate the complete and transparent reporting of RCTs and subsequently aid in their critical appraisal and interpretation. Over time, evidence of the impact of CONSORT has accumulated. It is one of the most widely cited scientific contributions of all time (over 5,300 citations, not including self-citation). CONSORT has also been rated as one of the major milestones in health research methods over the last century by the Patient-Centered Outcomes Research Institute (PCORI) [6]. The most recent version, CONSORT 2010 is among the top 1% of article-level content contained in the Public Library of Science (http://www.plos.org/). Its use has been endorsed by international organisations of editors such as the International Committee of Medical Journal Editors (ICMJE), the World Association of Medical Editors (WAME), and the Committee On Publication Ethics (COPE). Over 600 general and specialty journals currently endorse the CONSORT Statement [7].

‘Endorsement’ of CONSORT by a journal is defined as any of the following situations, which imply that the CONSORT Statement is, at least, in principle incorporated into the editorial process of the journal: (a) journal editorial statement endorsing the CONSORT Statement: either the flow diagram, the checklist or both; (b) requirement or recommendation in journal’s ‘Instructions to Authors’ to follow CONSORT when preparing their manuscript; or (c) requirement for authors to submit a CONSORT checklist and/or flow diagram with their manuscript. At this time, information with regards to the regulation and enforcement of use and adherence to CONSORT by editors is too sparse to incorporate in this review. Complete reporting was assessed by comparing the proportion of RCTs adhering to individual CONSORT items, blinding subgroups or by total scores across CONSORT checklist items termed ‘Total sum score’.

Along with the publication of the 2001 version of CONSORT, Moher and colleagues reported an evaluation of the CONSORT checklist [8]. The authors reported that the completeness of reports of RCTs in three CONSORT endorsing journals was higher than one non-endorsing journal. Since then, additional evaluations have been published which assess the influence of CONSORT either directly or indirectly. In 2006 a systematic review identified eight evaluations that assessed the completeness of reporting in medical journals that did or did not formally endorse CONSORT [9]. Despite methodological weaknesses of included evaluations, the 2006 systematic review found that the use of CONSORT may improve the quality of reporting of RCTs.

## Objective

This paper provides a comprehensive summary of the 91-page report of the updated systematic review assessing the influence of CONSORT, published in the Cochrane Library [10]. The objective of the update was to assess whether journal endorsement of the CONSORT Statement is associated with improved completeness of reporting of RCTs.

## Methods

Electronic database searches were performed to update the 2006 systematic review for the time period January 2005 to March 2010, inclusive, searched on 27 May 2010. The end search date was purposely selected to exclude the date of publication of the most recent CONSORT Statement (CONSORT 2010) as it was not expected it to have been in use for a sufficient period to have produced evaluations.

MEDLINE (via OVID), EMBASE (via OVID), the Cochrane Methodology Register and the Cochrane Database of Systematic Reviews, both using the Wiley interface, were searched using a comprehensive strategy [10]. The following citation indices were also searched using the ISI Web of Knowledge interface: The Science Citation Index, Social Science Citation Index, and Arts and Humanities Citation Index. Electronic records were stored and managed online through systematic review data management software, DistillerSR® [11].

Studies were included in the review if they evaluated the completeness of reporting of RCTs, and could be included in any of the following comparison groups, by assessing the: (1) completeness of reporting assessed in RCTs published in CONSORT-endorsing journals compared to non-endorsing journals; (2) completeness of reporting of RCTs published in CONSORT endorsing journals before and after endorsement; or (3) completeness of reporting of RCTs before and after the publication of the 1996 or 2001 CONSORT Statement. Studies were not excluded based on language of publication or validity assessment. The CONSORT checklist is being endorsed by non-medical journals, typically in veterinary trials [12], such ‘non-human’ trial evaluations were not eligible for this review.

When the eligibility of studies was either unclear or the data reported were insufficient to categorize the study into at least one of the reviews’ pre-specified comparison groups, corresponding authors were contacted for clarification and invited to submit unpublished data. This enabled judgment about study eligibility and, if eligible, provided data which were extracted for meta-analyses, when possible. For example, if the citations for RCTs included in an evaluation were provided, review authors could determine endorsement status of their publishing journals at the time of RCT publication by checking a journal’s Instructions to Authors and/or contacting editors of journals. A journal was considered an endorser if it had endorsed CONSORT at least months prior to publication of the evaluated RCT.

Title, abstract and full text screening of potentially relevant records was completed by two independent reviewers. One reviewer extracted general study characteristics of included studies, with complete verification by a second reviewer. Data on the completeness of reporting were extracted by one reviewer; a second reviewer verified accuracy of a 10% random sample. Discrepancies were resolved by discussion between the two reviewers or arbitration with a third member of the research team, when necessary. No modifications to the extracted data were made post-verification. Validity of included studies was assessed by one reviewer with complete verification by a second reviewer using pre-specified criteria [10].

Data on 27 outcomes were collected to estimate completeness of reporting of RCTs in included evaluations. These were: any of the 22 items on the 2001 CONSORT checklist, any of four additional sub-items for blinding (outcome assessor, intervention, patients and data analyst), or ‘Total Sum Score’ (that is, an aggregate score of some or all CONSORT checklist items). While data extraction was based on items of the 2001 CONSORT checklist, where studies evaluated the 1996 checklist, analyses were sub-grouped by checklist year since some checklist items were known to have undergone substantial modification in the 1996 to 2001 CONSORT update. These items were: ‘Title and Abstract’, ‘Outcomes’, ‘Sample Size’, ‘Participant Flow’ and ‘Numbers Analysed’ [10].

Relative proportions of RCTs adequately reporting any of the 27 outcomes were extracted from included studies. In addition, if quality of RCTs within included evaluations was assessed using a quality assessment tool however measured (for example, Jadad scale [13]), these data were extracted.

Relative risks (RR) and 99% confidence intervals were calculated to determine effect estimates for each outcome across evaluations. Reported mean scores across some or all checklist items, described as a ‘Total Sum Score’ were also pooled where a standardised mean difference (SMD) greater than zero indicates more complete reporting among RCTs published in CONSORT-endorsing journals.

## Results

**Figure 1:**
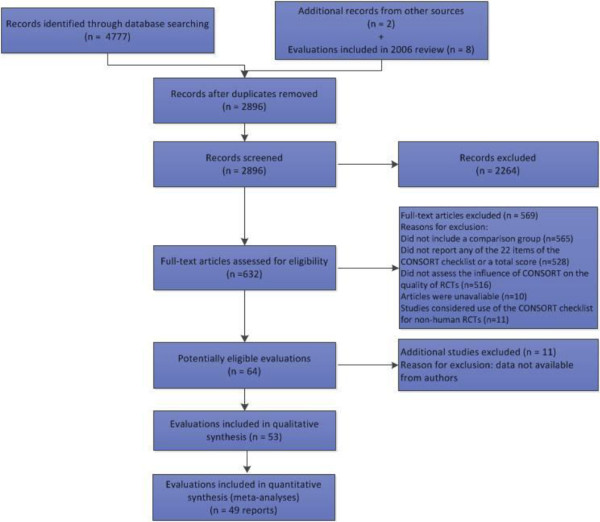
PRISMA flow diagram.

The electronic searches retrieved a total of 4,777 records; two additional reports were identified. In addition, eight reports were included in the 2006 systematic review. Duplicate records were removed, and the titles and abstracts of the remaining 2,896 records were screened yielding 632 potentially relevant studies. Full text screening identified 53 reports of 50 evaluations (Figure 1). Two of the three comparison groups include evaluations where more information is known about endorsement of CONSORT as a more direct intervention. Subsequently, we report the findings of these two comparison groups, ‘CONSORT endorsing journals compared to non-endorsing journals’ and ‘CONSORT endorsing journals before and after’. Please refer to the Cochrane review for results of the third comparison group, cross-sectional samples of RCTs before and after the publication of the CONSORT Statement.

### CONSORT-endorsing journals compared to non-endorsing journals

Twenty-nine (of 53) evaluations were eligible for this comparison group. Across the 27 outcomes the number of studies per meta-analysis varied (median (IQR1, IQR3), 6 [5,8]). Adequate reporting of the method of ‘Allocation Concealment’ and the description of flow of participants through the trial, ‘Participant Flow’, had the largest number of included studies (*n*=16), evaluating adequacy of reporting in 2,396 and 2,140 RCTs, respectively.

**Figure 2:**
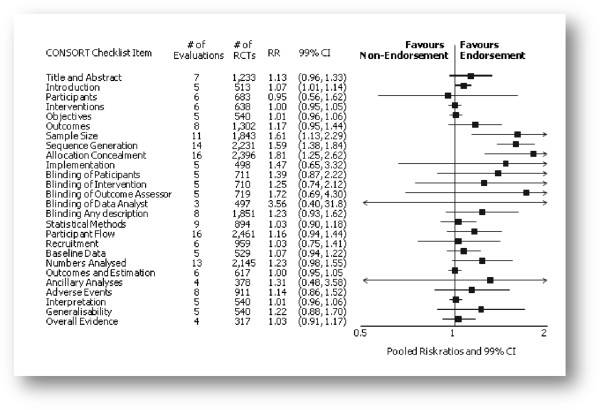
Pooled relative risks across assessed 2001 CONSORT checklist items with 99% confidence intervals for primary comparison, adherence of RCTs published in CONSORT-endorsing journals compared to RCTs published in CONSORT non-endorsing journals.

Of 27 outcomes evaluated, 25 had effect estimates indicating a relatively higher proportion of completely reported RCTs published in CONSORT-endorsing journals compared to non-endorsing journals. Of these, five were statistically significant at the 1% level (Figure 2); adequate details of method of ‘Allocation Concealment’ RR = 1.81 (1.25, 2.61) (16 evaluations, 2,396 RCTs, I^2^= 75%). This suggests that 81% more RCTs published in CONSORT-endorsing journals described allocation concealment more completely compared to those published in non-endorsing journals.

Other outcomes which resulted in statistically significant effects, favouring the completeness of reporting in CONSORT-endorsing journals were providing an adequate scientific explanation and rationale detailed in the ‘Introduction’ of the trial, RR = 1.07 (1.01, 1.14) (five evaluations, 513 RCTs, I^2^ = 0%), adequate description of how ‘Sample Size’ was determined RR = 1.61 (1.13, 2.29) (11 evaluations, 1,843 RCTs, I^2^ = 76%), and adequate description of the method used for ‘Sequence Generation’ RR = 1.59 (1.38, 1.84) (14 evaluations, 2,231 RCTs, I^2^ = 24%). Seven included studies evaluating 560 RCTs contributed to a statistically significant pooled effect in favour of CONSORT for ‘Total Sum Score’: SMD = 0.68 (0.38, 0.98) (I^2^ = 0%). This indicates that when adequate reporting was summarised across all CONSORT items, RCTs published in CONSORT-endorsing journals were more completely reported than RCTs published in non-endorsing journals.

Precise details of ‘Interventions’, CONSORT checklist item four, were equally reported in endorsing and non-endorsing journals, RR=1.0 (0.95, 1.05) (six evaluations, 638 RCTs, I^2^ = 0%), and eligibility criteria for trial ‘Participants’ was reported in six evaluations assessing a total of 683 RCTs, and resulted in an effect estimate less than 1.0, RR= 0.95 (0.56, 1.62). This suggests that the relative completeness of reporting for this item is slightly less in CONSORT-endorsing journals compared to non-endorsing journals.

Eight studies were not strictly compliant with the definition of a CONSORT-endorsing journal used in this review. Sensitivity analyses showed that only one outcome (of 27), although only minimally different, differed when evaluations that did not directly meet our definition of endorsement were excluded. Completeness of reporting of the scientific rational and background in the ‘Introduction’, was adjusted from RR = 1.07 (1.01, 1.14) to 1.05 (0.87, 1.27).

### CONSORT-endorsing journals before and after CONSORT endorsement

**Figure 3:**
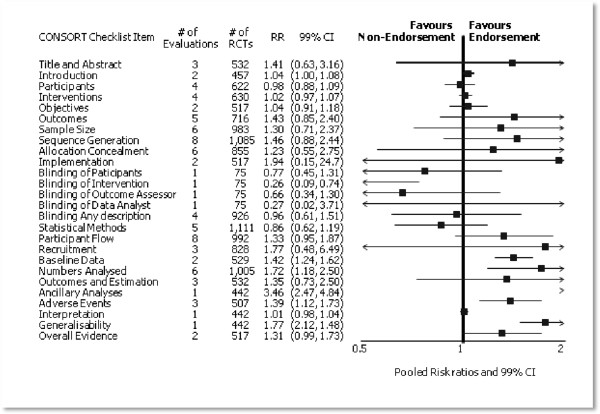
Pooled relative risks across assessed 2001 CONSORT checklist items with 99% confidence intervals comparing the adherence of RCTs published in CONSORT-endorsing journals before and after endorsement.

Eleven (of 53) evaluations assessed journals that endorse the CONSORT Statement and presented RCT completeness of reporting of at least one CONSORT item before and after the journal’s date of endorsement of CONSORT. The number of RCTs assessed per outcome had a median (IQR) of 532 (512, 919). The number of reported CONSORT checklist items varied over evaluations, with a median (IQR) of 3 (2, 5). Adequate reporting of the method of ‘Sequence generation’ and the flow of participants through the trial, ‘Participant Flow’, were both reported in eight evaluations. For 15 of 27 outcomes data were reported in fewer than five evaluations. The results across all outcomes in this comparison are presented (Figure 3).

## Discussion

Serious systemic problems exist in how research is reported. This is a wasteful use of already limited resources that fund health research [14]. This issue might reflect several factors including inadequate conduct, sloppiness in reporting, and quite possibly an inability of researchers to clearly and transparently inform readers about the methods and findings of their research [15]. The problem is endemic known to affect many, if not all, areas of healthcare research [16-18]. Systematic reviews evaluating the effectiveness of health interventions typically rely on reports of RCTs as their primary source of evidence [19]. However, the inadequate reporting of essential elements in these reports hinders the systematic review process, often leaving reviewers unable to make definitive conclusions [20].

This review suggests that RCTs which report their findings using the CONSORT statement as guidance may produce more complete RCT reports. While this review provides some important insight into the impact of CONSORT on completeness of reporting it is not without limitations. Although the search was completed more than 2 years ago we do not believe that any new evaluations would alter the present results as the included number of evaluations is large and includes data from over 16,600 trials. The majority of CONSORT evaluations are small and very large studies with dramatic effect estimates would need to be included to modify the current results. Similarly, newer evaluations may evaluate the CONSORT 2010 checklist which are ineligible for this systematic review. None of the included evaluations utilized an experimental design (that is, randomised trial) and as such are subject to the influence of confounding, in particular, potential improvement in completeness of reporting over time as well as discrepant editorial policies between journals. The inclusion criteria for this review were broad and variability in methodology, field of interest and validity between evaluations was considerable. The use of 99% confidence intervals, intended to estimate conservative effects given the questionable validity and heterogeneity of included studies, aims to offer readers with more confidence in the findings of this review, especially those of statistical significance.

There are inconsistencies in how journals implement reporting guidelines when reports of RCTs are submitted for publication [21,22]. Since editorial procedures are not consistently available online, this review was unable to assess journal implementation of CONSORT endorsement (that is, verification by the journals’ editorial team of author adherence to CONSORT). Anecdotally, there is variability between how verification for CONSORT adherence by authors factors into the editorial decision-making process (if at all) between journals. Editors may want to use these results develop explicit statements about their journals’ endorsement of CONSORT, and other reporting guidelines, in their ‘Instructions to Authors’, and optimally to recommend submission of the CONSORT checklist and flow diagram at the time of manuscript submission [23]. It is likely that active endorsement policies by journals will lead to more complete, clear and transparent publications, ultimately increasing the usability of research reports to make decisions about healthcare treatment and services.

## Conclusions

The results of this review suggest that journal endorsement of CONSORT may benefit the completeness of reporting of RCTs they publish. No evidence suggests that endorsement hinders the completeness of RCT reporting. However, despite relative improvements when CONSORT is endorsed by journals, the completeness of reporting of trials remains sub-optimal. Journals are not sending a clear message about endorsement to authors submitting manuscripts for publication. As such, fidelity of endorsement as an ‘intervention’ has been weak to date. Journals need to take further action regarding their endorsement and implementation of CONSORT to facilitate accurate, transparent and complete reporting of trials.

All CONSORT guidance documents including the 2010 checklist, flow diagram, extension statements and other resources are freely available from the CONSORT website [24] and in numerous open-access publications. Their consistent use and adherence by authors and journals has the potential to revolutionize the reporting and subsequent usefulness of RCT reports.

## Endnote

^a^This review is an abridged version of a Cochrane Review recently published in the Cochrane Database of Systematic Review 2012, Issue 11, DOI: 10.1002/14651858.MR000030.pub2. (See http://www.thecochranelibrary.com for information). Cochrane reviews are regularly updated as new evidence emerges and in response to feedback, and Cochrane Database of Systematic Reviews should be consulted for the most recent version of the review.

## Competing interests

Three team members (DM, DGA and KFS) form the executive membership of the CONSORT Group and have led the development of the CONSORT Statement since inception. All three are also members of the EQUATOR Network executive committee. One team member (LS) is CONSORT research staff, for which partial salary support is provided by a grant from the Medical Research Council, United Kingdom.

## Authors' contributions

LT and LS drafted the text of this manuscript based on research carried out and described in the associated Cochrane review. All authors (LT, LS, DM, DGA, KFS) read and approved the final manuscript.
